# A Half-Century Studies on Epidemiological Features of Ancylostomiasis in China: A Review Article

**Published:** 2019-09

**Authors:** Rui LI, Jie GAO, Lingxi GAO, Yajun LU

**Affiliations:** 1. Department of Clinical Pathogen Biology and Laboratory, School of Tropical Medicine and Laboratory Medicine, Hainan Medical University, Haikou, China; 2. Laboratory of Tropical Translational Medicine of Ministry Education, Haikou, China; 3. Department of Microbiology, School of Medicine, Guangxi Medical University, Nanning, China

**Keywords:** Hookworm, Epidemiological features, Hospital cases, Misdiagnosis

## Abstract

**Background:**

Ancylostomiasis is a prevalent and global parasitic disease, including China. A systematic review is significant to understand the epidemiological features of hookworm and provide guidance for prevention and treatment.

**Methods:**

We systematically searched academic databases and assessed 944 papers published from 1955–2015 to establish the comprehensive analysis of prevalence of hookworm disease in China. We searched Chinese databases, including CNKI, Wanfang and VIP, for literature with the subject word “Ancylostomiasis and hookworm”. The data were analyzed with SPSS 19.0 software using Spearman correlation analysis. Results were statistically significant for a *P*-value of <0.01.

**Results:**

The search yielded 532,151 cases from epidemiological investigation and 7294 cases based on hospital diagnosis. Hookworm infection was highest (15.83%) in Fujian province, with high rates also found in East China, Southwest China, Central China and Southern China and lower rates in Northwest China, North China and Northeast China. In terms of occupation, farmers had the highest proportion of infections (72.54%). There was no correlation between epidemiological investigations and hospital-diagnosed cases. However, there was significant positive correlation between hospital-diagnosed cases and misdiagnosed cases. The proportion of hospital-misdiagnosed cases was 32.80%.

**Conclusion:**

Ancylostomiasis is a serious public health problem that negatively influences health and hinders socioeconomic development. Positive measures are required by both health services and individuals to prevent and control hookworm disease.

## Introduction

The Ancylostomatidae worms are collectively known as hookworm and comprise two species: *Ancylostoma duodenale* and *Necator americanus* (1). Hookworm adults mainly live in the human small intestine (2). Their eggs are excreted to the environment in feces and gradually develop in vitro into filariform larvae in warm, moist loose soil with sufficient oxygen (3). Obvious thermotropism and hygrotropism are demonstrated by filariform larvae, and in soil they are stimulated by body surface temperature to strengthen their mechanical puncturing movement and enzymatic action. They are able to puncture the skin actively through hair follicles, sweat glands and skin wounds (4), and can also invade humans through perioral and transplacental routes.

Of the intestinal nematodes, hookworm significantly impairs the health of infected individuals (5). The adult worms damage the intestinal mucosa causing gastrointestinal dysfunction, which can result in long-term chronic blood loss, leading to severe anemia (6). Ancylostomiasis is widely globally distributed (7), but mainly found in tropical and subtropical regions (8). In China, the vast rural areas south of the Yellow River are the main endemic regions (9). Two national surveys on parasitic diseases have been conducted in China, the first from 1988 to 1992 and the second from 2001 to 2004 (10–11). The first survey indicated that 194 million people were infected with hookworm, which translated as 17.2% of the total population. According to the second survey, the National Survey of the Major Human Parasitic Diseases in 2005, the number of people infected with hookworm was about 39.3 million, which was 6.12% of the total population.

Our study reviews hookworm infection using information published on the database over the past six decades. We collected information on the epidemiological characteristics of the disease, including infection status, risk factors, high-risk groups and misdiagnosis. An analysis was performed to characterize the epidemiology and infection trends, to guide diagnosis, prevention and control leading to the long-term aim of disease elimination.

## Methods

### Article selection

We searched Chinese databases, including CNKI, Wanfang and VIP, for literature with the subject word “hookworm”. We also searched literature with both the subject words “China and hookworm” in the PubMed, Ovid, SpringerLink, ScienceDirect and Embase databases. Searched literature ranged in date from 1955 to 2015. Full-text articles found were retrieved and selected for assessment according to inclusion and exclusion criteria.

### Inclusion and exclusion criteria

Articles on hookworm infection that were epidemiological investigations and hospital case reports were included. There were no restrictions to literature type.

The following exclusion criteria were applied: 1) duplicates were excluded, with the more recently published article or the one with more detailed data retained; 2) articles lacking numerical data on hookworm infection were excluded; and 3) articles reporting molecular biology studies, animal experiments, drug treatments, pathogenic mechanisms or hookworm prevention were excluded.

### Factor analysis

We analyzed general infection status, occupation and regional distribution, and clinical data including provincial distribution, reason for admission, clinical characteristics, organs affected, examination methods, infection progression over 5-year periods and misdiagnosis. Cases with initially inaccurate, delayed or incomplete diagnosis causing delayed or untargeted treatment were considered to be misdiagnosed.

### Statistical analysis

Excel 2007 was used to establish a database, and SPSS 19.0 software was used for statistical analysis. The results were considered statistically significant if the *P*-value was <0.01.

The purpose of the study was to investigate the epidemiological characteristics of hookworm disease in China. Data analysis included the evaluation of epidemiological data and the analysis of hospital diagnostic data. The specific objective was to evaluate for correlation between the number of positive cases and hospital-diagnosed hookworm disease cases, in order to understand the relationship between hookworm infection and the number of patients. Spearman correlation analysis was used to evaluate the correlation between these two discrete variables (12).

To evaluate the accuracy of the diagnosis of hookworm disease in hospital, the correlation between the number of hospital diagnostic cases and the number of misdiagnosed cases was evaluated. These two variables were discrete and so Spearman correlation analysis was used.

Correlation analysis using the Spearman’s coefficient measures the strength and direction of the monotonic association between two discrete variables (13). A monotonic relationship is one that does either of the following: (1) as the value of one variable increases, so does the value of the other variable; or (2) as the value of one variable increases, the other variable value decreases (14).

## Results

### Article evaluation

**Fig. 1:**
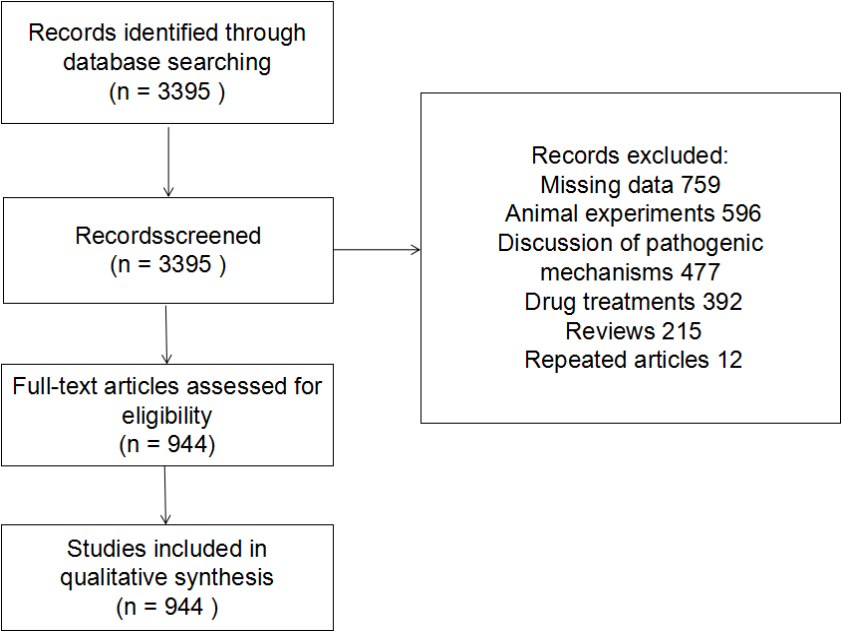
Flow diagram of selection process (n=number of articles; A total of 944 articles were included in the final analysis according to inclusion and exclusion criteria from 1955 to 2015.)

We retrieved a total of 3395 articles from the databases. Of these, 2451 did not meet the study inclusion criteria (i.e., missing data: 759 articles, animal experiments: 596, discussion of pathogenic mechanisms: 477, drug treatments: 392, reviews: 215, repeated articles: 12). A total of 944 articles were included in the final analysis (Fig. 1). Of these, 385 articles featured hospital diagnosis of hookworm and 559 articles reported on epidemiological investigations. In addition, 110 articles were reports on misdiagnosis of hookworm infection in hospital.

### Hookworm infection status according to occupation

The 944 articles conforming with the inclusion criteria totaled to 539 445 cases of hookworm infection, including 532 151 cases (98.65%) from epidemiological investigations and 7294 patients (1.35%) diagnosed at hospitals.

Infected cases were divided by occupation into farmers, students, preschool children, laborers, the elderly, prisoners, soldiers, fishermen, police and occupation not detailed. Analysis showed that farmers were a high-risk group for ancylostomiasis (Table 1).

**Table 1:** Infection status according to occupation (n=539 445)

***Occupation***	***Epidemiology investigation***		***Hospital diagnosis***		***Total (Case)***
	***Case***	***Percent (%)***	***Case***	***Percent (%)***	
Farmers	388122	72.93	3215	44.08	391337
Students	8541	1.60	5	0.07	8546
Preschool children	2577	0.48	668	9.16	3245
Laborers	2164	0.41	485	6.65	2649
The elderly	1887	0.35	0	0.00	1887
Prisoners	716	0.13	0	0.00	716
Soldiers	665	0.12	2	0.03	667
Fisherman	63	0.01	0	0.00	63
Police	8	0.00	0	0.00	8
Unstated	127408	23.94	2919	40.01	130327
Total	532151	100.00	7294	100.00	539445

### Geographical distribution

**Fig. 2:**
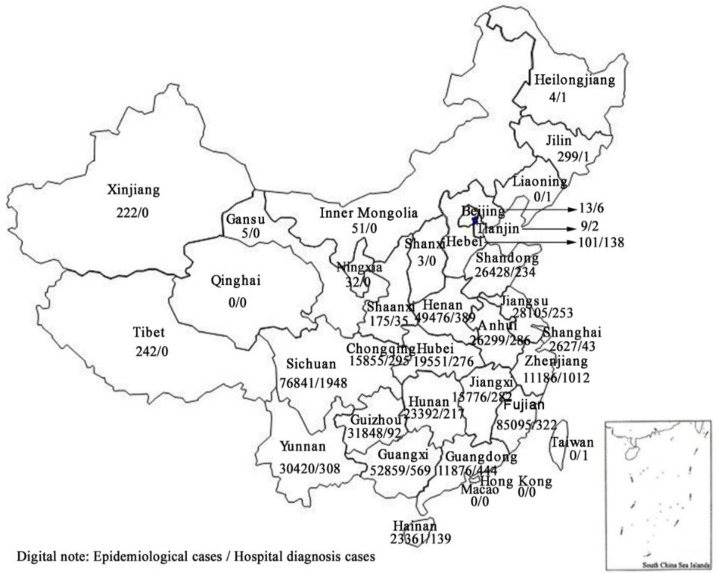
Distribution of epidemiological and hospital cases by province

China is officially divided into seven areas. The hookworm infection rates according to epidemiological investigation for each area, from high to low, were East China: 36.69%, Southwest China: 29.26%, Central China: 17.30%, South China: 16.54%, Northwest China: 0.09%, North China: 0.06% and Northeast China: 0.06% (Table 2). Thirty provinces and administrative regions had hookworm reports; three regions did not have any reports: Qinghai, Hong Kong and Macao (Fig. 2).

**Table 2:** Epidemiology survey and hospital-diagnosed cases by area and province (n=539 445)

***Area***	***Province***	***Epidemiology investigation (case)***	***Hospital diagnosis(case)***	***Total (case)***	***Area total(case)***
East	Fujian	85095	322	85417	197949
	Jiangsu	28105	253	28358	
	Shandong	26428	234	26662	
	Anhui	26299	286	26585	
	Jiangxi	15776	282	16058	
	Zhejiang	11186	1012	12198	
	Shanghai	2627	43	2670	
Southwest	Sichuan	76841	1948	78789	157849
	Guizhou	31848	92	31940	
	Yunnan	30420	308	30728	
	Chongqing	15855	295	16150	
	Tibet	242	0	242	
Central	Henan	49476	389	49865	93301
	Hunan	23392	217	23609	
	Hubei	19551	276	19827	
South	Guangxi	52859	569	53428	89248
	Hainan	23361	139	23500	
	Guangdong	11876	444	12320	
Northwest	Xinjiang	222	0	222	469
	Shanxi	175	35	210	
	Ningxia	32	0	32	
	Gansu	5	0	5	
	Qinghai	0	0	0	
North	Hebei	101	138	239	323
	Inner Mongolia	51	0	51	
	Beijing	13	6	19	
	Tianjin	9	2	11	
	Shanxi	3	0	3	
Northeast	Jilin	299	1	300	306
	Heilongjiang	4	1	5	
	Liaoning	0	1	1	

Hospital-diagnosed cases were reported from 24 provinces and administrative regions. Tibet, Xinjiang, Ningxia, Gansu, Qinghai, Inner Mongolia, Shanxi, Hong Kong and Macao did not report any hospital-diagnosed cases. Sichuan province had the most hospital-diagnosed cases.

### Clinical characteristics of hospital-diagnosed cases

Of the 7294 hospital-diagnosed cases, 3727 were male (51.10%) and 3567 female (48.90%). The youngest patient was 3-days old and the oldest 89 years. The shortest disease duration was 5 days, and the longest, owing to misdiagnosis, 15 years.

Ancylostomiasis is caused by risky dietary and lifestyle habits, such as eating unclean fruit and vegetables, working barefoot in fields, poor sanitation (particularly the mismanagement of fecal matter) and contact with soil polluted with filariform larvae. Hookworm infection pathways are mainly percutaneous, perioral and transplacental, the most common being percutaneous infection. Among the 7294 hospital-diagnosed cases, 6821 (93.52%) were infected cutaneously, 51 (0.70%) were infected transplacentally and 4 (0.05%) were infected by the oral route. A total of 418 (5.73%) cases did not have the infection route reported in the articles.

### Affected organs and diagnostic method for hospital-diagnosed cases

There were definite reports on affected organs for 3847 hospital-diagnosed cases. The host’s intestinal tract was the most commonly affected, mainly the duodenum, followed by multiple organs affected.

Hookworm adults parasitize the human small intestine and their eggs are excreted with the host feces; thus host feces is the material used for clinical examination (15). The common methods of analysis for hookworm infection are the direct smear method, saturated saline float method, modified Kato-Katz method, precipitation method and hookworm larva culture method (16). It is also regarded as evidence of diagnosis if worms are found by endoscopy, gastroscopy or other gastrointestinal endoscopy methods.

The 532 151 cases from epidemiological investigation were all diagnosed by stool examination. Of the hospital-diagnosed cases, 3328 (45.63%) were diagnosed by stool examination, 3314 (45.43%) by gastrointestinal endoscopy and the remaining cases by the above two methods.

### Misdiagnosis of hospital-diagnosed cases

There were 2391 misdiagnosed cases among the hospital cases, including 1333 male (55.75%) and 1058 female (44.25%), a total proportion of 32.78% of all hospital-diagnosed cases. The main causes of misdiagnosis were the diversity of clinical symptoms and lack of defining symptoms. Of the misdiagnosed cases, 1605 patients presented with anemia, 1086 with abdominal pain, 1069 patients with tarry stools, 307 with dizziness or weakness and 101 with gastrointestinal bleeding. Of the misdiagnosed cases, 452 were misdiagnosed as gastrointestinal bleeding, 209 as anemia including 42 cases with iron deficiency anemia, 23 cases as canker, 12 cases as bronchial asthma, 3 as acute leukemia, 1 as chronic nephritis, 1 as rheumatic heart disease, 1 as chronic bacillary dysentery and 1 as schizophrenia.

**Fig. 3:**
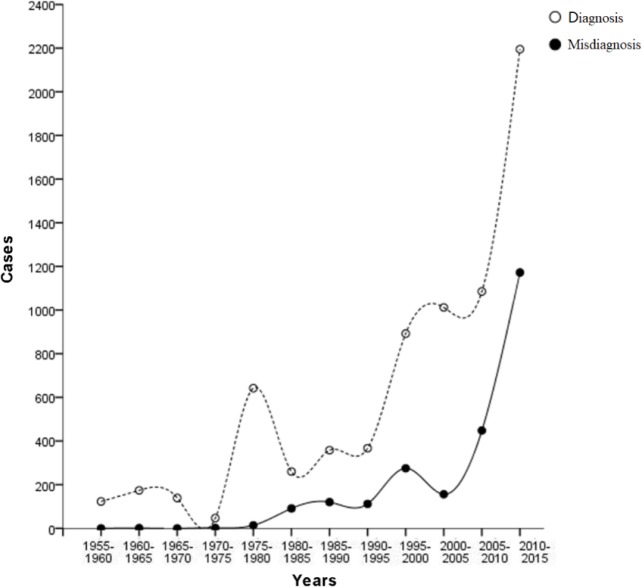
Hospital-diagnosed and misdiagnosed case numbers for 5-year periods from 1955 to 2015. (Spearman correlation analysis: r=0.935, *P*<0.01)

The correlation between the numbers of misdiagnosed cases and confirmed hospital-diagnosed cases was analyzed in 5-year periods. Misdiagnosis showed an increasing trend except for the years 2000–2005. In general, there was significant positive correlation between the numbers of hospital-diagnosed cases and misdiagnosed cases for the period 1955–2015 (r=0.935, *P*<0.01) (Fig. 3).

### Progression of hookworm infection

According to analysis by 5-year phases from 1955 to 2015, epidemiological infections were highest from 1990–1995. They showed an increasing trend during 1955–1960, 1985–1990 and 2005–2010. On the contrary, infections showed a downward trend during 1960–1965, 1980–1985 and 1995–2000.

**Fig. 4:**
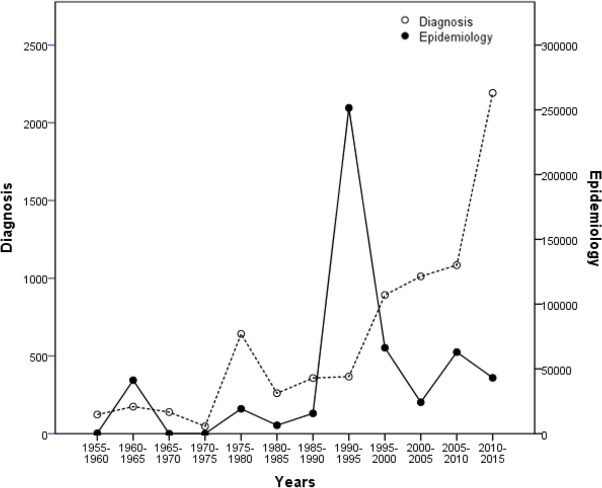
Epidemiological investigation and hospital-diagnosed hookworm cases for 5-year periods from 1955 to 2015. (Spearman correlation analysis: r=0.091, *P*=0.778)

Hospital-diagnosed cases displayed an increasing trend year by year, and increased at a faster rate during 1975–1980 and 2005–2010 than in other phases (Fig. 4). There was significant positive correlation between the number of epidemiological investigation cases and hospital-diagnosed cases (r=0.091, *P*=0.778).

## Discussion

Ancylostomiasis, schistosomiasis, malaria, filariasis and leishmaniasis were listed as five major parasitic diseases in the early Peoples’ Republic of China. Ancylostomiasis is widely prevalent in China (17) and is a public health problem with a considerable negative influence on health, hindering socioeconomic development (18). The pathogenic mechanism of both hookworm species is similar (19–20), with their eggs developing into infective larvae in vitro (21), which then puncture human skin (22) to migrate and reach the small intestine (23). Hookworm eggs were express as eggs per gram of feces. (EPG). Hookworm infections were designated as light (1–399 EPG), moderate (400–3000 EPG) or heavy> 3000 EPG. Hookworm species (*A. duodenale* and *N. americanus*) was determined by morphological identification of third-stage larvae which were successfully reared from eggs by coproculture from people. For identification of subjects with mixed or single hookworm infections, at least 100 third-stage larvae were examined (24). Hookworm adults usually live in the upper small intestine and damage intestinal mucosa by biting with their broad teeth. Hookworm can cause *Ancylostoma* dermatitis when they puncture skin, as well as anemia and other digestive tract symptoms.

The spread of hookworm is associated with the natural environment, farming methods, lifestyle habits and other factors (25), with filariform larvae needing suitable temperature, humidity and soil nutrition to survive in the external environment (2). Thus, hookworm infection prevalence differs by region (26). According to this review, hookworm infection rates were higher in East China, Southwest China, Central China and Southern China than in Northwest China, North China or Northeast China. Infection rates were higher in Fujian (15.83%), Sichuan (14.61%) and Guangxi (9.90%) provinces and other southern areas. However, Beijing (0.004%), Tianjin (0.002%), Liaoning (0.0002%) and other northern provinces had lower hookworm infection rates. In general, the provinces with higher hookworm infection rates have abundant rainfall, mild climates and loose fertile soil, highly suited to the growth and spread of hookworm larvae (27). Their communities are more likely to use untreated human feces to directly irrigate and fertilize crops (28), and farmers are more likely to labor in fields barefoot. Poor dietary lifestyle habits such as eating unwashed fruit and vegetables (29) contribute with these other behaviors to human contact with soil contaminated by filariform larvae and thus infection with hookworm (30). This makes farmers a high-risk group for ancylostomiasis, consistent with previous reports. The data also suggested that infants were infected from diapers and clothing polluted by hookworm larvae, as well as by sleeping in baskets and playing in fields (31). These are actions that may result in direct and indirect hookworm infection. Other infant infections may be caused by eating unwashed food or through the placenta (32).

Hookworm disease is easily misdiagnosed because clinical symptoms are non-specific. Most patients lack obvious symptoms and only show gastrointestinal symptoms such as epigastric discomfort, nausea, vomiting, diarrhea and constipation. Patients may have gastrointestinal bleeding and anemia (33–34). This review showed that hookworm disease may not only involve the intestinal tract, including the duodenum, jejunum, stomach, ileum, cecum, colon and rectum, but may also involve other parts of the abdomen, the pancreas or multiple organs. The proportion of hospital cases misdiagnosed in our study was 32.78%. Hookworm adults cause focal hemorrhage and small ulcers by biting intestinal mucosa with broad, hooked teeth. The hemorrhaging of the digestive tract, ulceration and chronic hemorrhagic anemia that can be caused by the biting of hookworm adults (35) is easily misdiagnosed as gastrointestinal hemorrhage and digestive ulcer, for three important reasons. First, the clinical symptoms of ancylostomiasis are atypical and patients and physicians have difficulty identifying it. Infections can be asymptomatic or present with only mild symptoms such as mild epigastric discomfort, pallor, vomiting, diarrhea or mild anemia (35). Patients presenting as such have a longer course of disease and are easily misdiagnosed. Second, clinicians may not have experience of managing hookworm infection, believing that gastrointestinal bleeding is caused by mucosal inflammation, erosion, ulcer, tumor or vascular lesions of the digestive tract. Clinicians may consider hematological or heart disease when the main presentation is anemia (36) and few would consider hookworm infection. Third, if a single examination only is conducted, missed diagnosis or misdiagnosis are more likely. A considerable proportion of the hospital-diagnosed cases in our study received other examinations prior to stool examination. Clinicians should combine stool examination with gastrointestinal endoscopy, which may include gastroscopy, colonoscopy, double-balloon enteroscopy or capsule endoscopy, in clinical diagnosis (37). Stool examination prior to gastrointestinal endoscopy may improve the diagnostic rate. Although hookworm disease can easily be confused with other diseases, medical treatment is highly effective (38–39), with pyrantel, levamisole, mebendazole and albendazole commonly used (40). Iron and vitamin C are also recommended as supplements to pharmacotherapy (41).

We conclude that the possibility of ancylostomiasis should be considered in a number of situations, including patients from rural areas with cryptogenetic gastrointestinal bleeding or long-term chronic hemorrhagic anemia (15); patients whose fecal occult blood is positive but with digestive tract cancer, ulcer and other common causes excluded by routine examination; and patients in frequent contact with soil such as farmers and miners (42). A careful medical history should be taken and routine stool examination applied (43). It may be necessary to screen more than once, combining the saturated saline float method, modified Kato-Katz method and gastrointestinal endoscopy, to improve detection rates (44) and decrease missed diagnosis and misdiagnosis rates.

Our results showed that incidence was highest between 1990 and 1995, and incidence determined by hospital diagnosis increased over the 5-year periods. We summarize the reasons for these results. First, living conditions have improved rapidly with greater socioeconomic development since the Reform and Open, which caused a decrease in hookworm infection rates at that time. Second, knowledge of and record-keeping on clinical cases of ancylostomiasis has improved (45); literature about ancylostomiasis was unusual before 1990, and it is likely that more published research on the disease will have improved knowledge (46). Third, health promotion has been strengthened in recent years (47). Fewer people work barefoot in fields and better hygiene and lifestyle habits have gradually developed. Moreover, health services have attached greater importance to the prevention and control of hookworm disease (48). Manure management should be standardized to reduce environmental pollution and the opportunity for hookworm eggs to infect soil (49). Finally, the high rate of hookworm infection in the 1990–1995 period may be related to floods during this time. Further investigation would assist in confirming these hypotheses.

## Conclusion

Prevention and control of hookworm disease should focus on strengthening publicity and encouraging the participation of communities. Harmless fecal fermentation methods, improvement of water supply and sanitation, environment beautification and new energy production methods will all reduce soil contamination from hookworm eggs. Personal protective measures also need strengthening, such as promotion of the use of protective clothing and boots to farmers, to avoid exposed skin contact with soil especially after rain. In summary, a focus on improved personal, dietary and environmental hygiene will assist in reducing the prevalence of ancylostomiasis.

## Ethical considerations

Ethical issues (Including plagiarism, informed consent, misconduct, data fabrication and/or falsification, double publication and/or submission, redundancy, etc.) have been completely observed by the authors.
